# Introduction to the Special Issue on “Role of Novel Imaging Technique in Brain Tumors”

**DOI:** 10.3390/cancers16030575

**Published:** 2024-01-30

**Authors:** Ali Nabavizadeh

**Affiliations:** 1 Center for Data-Driven Discovery in Biomedicine (D3b), Children’s Hospital of Philadelphia, Philadelphia, PA 19104, USA; ali.nabavizadeh@pennmedicine.upenn.edu; Tel./Fax: +1-215-662-6865; 2 Department of Radiology, Perelman School of Medicine, University of Pennsylvania, Philadelphia, PA 19104, USA

In recent years, significant strides have been made in the field of neuro-oncology imaging, contributing to our understanding and management of brain tumors. MRI continues to be the workhorse for the diagnosis of tumors, assessment of tumor burden, and monitoring of tumor size during and after treatment [1]; however, there has been increasing evidence on the added value of various molecular imaging approaches, especially amino acid PET in brain tumors [2,3]. The rise of advanced computing in the past decade has led to the birth of the field of radiomics, which has been applied in brain tumor diagnosis and grading, prognostication, response assessment, differentiating treatment effects from tumor recurrence, and the molecular classification of tumors (i.e., radiogenomics) [4]. Finally, recent efforts in the integration of tumor segmentation and other artificial intelligence (AI) tools with picture archiving and communication system (PACS) platforms will help to accelerate the translation of these techniques to the clinic [5,6].

This Special Issue aims to present a cross-section of the state of the art in this research area, highlighting several exciting developments in the field of neuro-oncologic imaging.

The study by de Godoy et al. [7] highlights the clinical potential of proton MR spectroscopy (1H-MRS) at 3 Tesla with optimized echo time (TE) in identifying IDH-mutant gliomas. The research emphasizes the significance of oncometabolite 2HG as a key biomarker and demonstrates the efficacy of both single-voxel and multi-voxel 1H-MRS at being able to predict IDH mutational status. Additionally, the study identifies Glx (glutamate + glutamine) and NAA (N-acetylaspartate) as being crucial in distinguishing IDH mutants from wild-type gliomas. The findings suggest that 1H-MRS, with an optimized TE (97 ms), offers a non-invasive and accurate method for detecting 2HG levels and understanding their interplay with other metabolites in infiltrative gliomas.

The second study by Langen et al. [8] explores the additive value of amino acid PET and advanced MRI in cerebral glioma diagnosis, emphasizing the promise of a multimodal approach. While both methods independently offer valuable information, the study emphasizes the potential of a multimodal approach for improved diagnostics. There is increasing evidence that the combination of amino acid PET and advanced MRI improves grading and molecular characterization in newly diagnosed tumors, while data concerning the delineation of tumor extent and biopsy guidance are limited. Simultaneous PET/MRI can be especially beneficial in cerebral glioma patients, by reducing the number of imaging visits and examination time, and even more beneficial in children, by reducing the radiation burden and the need for repeated anesthesia for two separate examinations.

Liquid biopsy takes center stage in the third study by Khalili et al. [9], offering a non-invasive method to capture molecular diversity in CNS tumors, complemented by imaging techniques. The review underscores the challenges posed by the limited presence and short half-life of these biomarkers, especially in CNS tumors which have the additional complexity of the blood–brain barrier (BBB) and blood–tumor barrier compared to other solid tumors. Integration with imaging techniques is proposed to provide a more accurate characterization of brain tumors by identifying factors affecting plasma cfDNA and ctDNA detection, such as imaging BBB permeability; such integration also added to the value of imaging, including PET imaging, radiomics, and liquid biopsy, to better characterize brain tumors.

The fourth study by Rezaeijo et al. [10] introduces a generative method using Generative Adversarial Networks (GANs) to translate between T2-weighted-FLAIR and T2-weighted MRI volumes. This study proposes a novel evaluation schema based on radiomic features. The authors demonstrate that generative methods can produce realistic results without significant changes in radiometric features. This method has the potential to aid clinicians and researchers in having access to a missing sequence when rescanning is not feasible.

Finally, the study by Kaur et al. [11] explores the quantification of peritumoral edema using PACS-based tools, revealing its feasibility and potential to predict treatment outcomes. The research underscores the significance of monitoring edema volume in patients with lung cancer metastasis after stereotactic radiosurgery, particularly given that alterations in edema volume may precede changes in the size of the tumor core. This study nicely showcases an example of how PACS-integrated segmentation tools can be incorporated into clinical practice for a more comprehensive treatment response assessment.

In conclusion, these studies collectively showcase the evolving landscape of neuro-oncology imaging, from non-invasive metabolic assessments to the integration of advanced imaging modalities and innovative approaches for volumetric evaluation and PASCS integration. These advancements hold promise for refining diagnostic accuracy, predicting treatment outcomes, and ultimately improving patient care in the realm of neuro-oncology. Further progress is contingent upon increasing the reproducibility and generalizability of AI models and improved interoperability with clinical care and clinical trial workflows [12]. In addition, increased utilization of generative AI approaches will provide necessary tools for image-to-image synthesis, capable of generating absent or suboptimal MRI sequences [13]. Finally, despite mounting evidence highlighting the enhanced benefits of amino acid PET imaging, its widespread use is hindered in many countries by the absence of regulatory approval and reimbursement, which need to be addressed [14].
